# Joint-line medialization after anatomical total shoulder replacement requires more rotator cuff activity to preserve joint stability

**DOI:** 10.1016/j.jseint.2020.11.010

**Published:** 2021-02-02

**Authors:** Anita Hasler, Elias Bachmann, Andrew Ker, Arnd F. Viehöfer, Karl Wieser, Christian Gerber

**Affiliations:** a Department of Orthopedics, University of Zürich, Balgrist University Hospital, Zürich, Switzerland; b Laboratory for Orthopedic Biomechanics, Orthopedic University Hospital Balgrist, Zürich, Switzerland

**Keywords:** Total shoulder arthroplasty, Joint-line, Medialization, Lateralization, Muscle forces, Instability ratio

## Abstract

**Background:**

The biomechanical effects of joint-line medialization during shoulder surgery are poorly understood. It was therefore the purpose of this study to investigate whether medialization of the joint line especially associated with total shoulder arthroplasty leads to changes in the rotator cuff muscle forces required to stabilize the arm in space.

**Methods:**

A validated computational 3-D rigid body simulation model was used to calculate generated muscle forces, instability ratios, muscle-tendon lengths and moment arms during scapular plane elevation. Measurements took place with the anatomical and a 2 mm and 6 mm lateralized or medialized joint line.

**Results:**

When the joint line was medialized, increased deltoid muscle activity was recorded throughout glenohumeral joint elevation. The rotator cuff muscle forces increased with medialization of the joint line in the early phases of elevation. Lateralization of the joint line led to higher rotator cuff muscle forces after 52° of glenohumeral elevation and to higher absolute values in muscle activity. A maximum instability ratio of >0.6 was recorded with 6 mm of joint-line medialization.

**Conclusion:**

In this biomechanical study, medialization and lateralization of the normal joint line during total shoulder arthroplasty led to substantial load changes on the shoulder muscles used for stabilizing the arm in space. Specifically, medialization does not only lead to muscular shortening but also to increased load on the supraspinatus tendon during early arm elevation, the position which is already most loaded in the native joint.

Performing total shoulder arthroplasty (TSA) is a reliable way of relieving pain and improving function in patients with osteoarthritis of the glenohumeral joint.^11^^,^^28^^,^^29^ It is well documented that malpositioning of the glenoid component during TSA and especially failure to restore glenoid component version are associated with inferior outcomes. This may result in posterior humeral head displacement and joint instability,^8^^,^^10^^,^^26^^,^^31^ eccentric loading of the glenoid component, and premature aseptic loosening.^13^^,^^20^^,^^24^^,^^35^

A number of methods have been used to restore glenoid version to normative values. Open wedge posterior glenoid osteotomy^34^ has been used for correction of posterior instability and eccentric reaming, bone grafting, and augmented prosthetic glenoid components for correction when performing shoulder arthroplasty.^27^ In correcting glenoid version, all of these methods can result in medialization or lateralization of the native glenohumeral joint line.

Without quantification, in TSA surgery, medialization is often accepted to restore normal version in a retroverted arthritic glenoid. Eccentric reaming of the anterior subchondral glenoid bone has been used to correct up to 15 degrees of glenoid retroversion; however, further reaming can lead to a scapular neck that is no longer large enough to support a prosthetic glenoid component.^9^^,^^12^^,^^18^ When standard glenoid components are used, such correction of retroversion by eccentric reaming results in substantial medialization of the joint line. In a finite element study of 39 shoulders, Sabesan et al confirmed that correcting an average glenoid retroversion of 20.9 degrees (+/− 10 degrees) required 8.3 mm (+/− 4.1 mm) medialization to correct the version to neutral.^33^ Similarly, implanting glenoid components in the presence of a B2 glenoid^39^ and during glenoid revision surgery can result in medialization of the joint line.

The effect of medializing the joint line on the function of muscles that cross the glenohumeral joint is less clear. One effect may be shortening of these muscles, as documented by Roche et al. This study found that medialization of the joint line after TSA resulted in shortening of the rotator cuff muscles.^32^ However, the relevance of this finding was not further studied. The fact that a muscle loses the potential to develop tension if it is shortened is known and uncontested since the description of the length-tension relationship of the muscle by Blix in 1892.^5^ Friedman et al^15^ stated that medialization of the joint line must have implications on the length-tension relationship of the rotator cuff muscles and thereby compromise the muscles ability to move and to maintain joint stability.

Another potential adverse effect of medialization and shortening of the rotator cuff muscles is degeneration of the muscle. Donohue et al recently documented that B3 glenoids and increased pathologic retroversion, both associated with medialization of the center of the humeral head, are associated with high-grade fatty infiltration of the rotator cuff muscles.^12^ Furthermore, severe fatty infiltration of the rotator cuff muscles has been shown to substantially influence outcome after TSA in long-term clinical studies.^42^

Adequate function of the rotator cuff muscles is critical to the outcome after TSA. In the literature, secondary rotator-cuff tears account for 7%-20% of complications after TSA^6^^,^^19^ and as per the 2018 Australian Orthopaedic Association National Joint Replacement Registry data, rotator cuff insufficiency is the most frequent cause of revision for TSA.^2^

The aim of this study was to investigate, in a 3-D computational model of a standard TSA featuring cocontractors, whether medialization or lateralization of the joint line alters the forces generated by the deltoid and rotator cuff muscles, as well as joint stability, while elevating the arm in space. We hypothesize that with increasing medialization of the joint line, a greater force will be required in the rotator cuff muscles to maintain elevation and joint stability. Furthermore, medialization of the joint line would result in greater forces required by the deltoid muscle to elevate the arm.

## Methods

**Figure 1:**
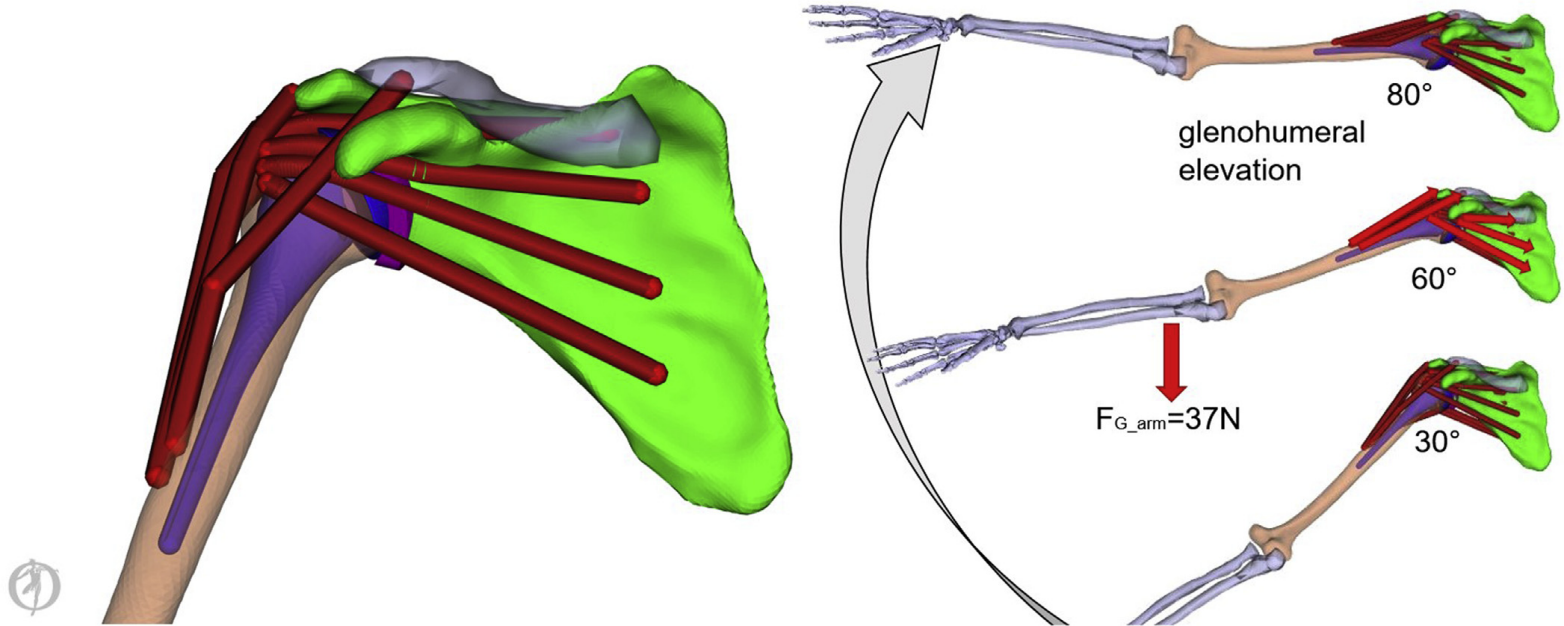
Modified OpenSim model from Holzbauer et al^23^ to simulate the rotator cuff and deltoid muscle forces in abduction between 30° and 80° in 2° steps. The rigid body simulation determines by loading a predefined motion file (elevation of the arm in scapular plane), the forces necessary to cause this particular motion. Based on performance criteria like minimizing the sum of squared muscle forces those equations of motions are solved.

To analyze the biomechanical influence of joint-line medialization, a rigid body shoulder model was used to calculate the required muscle and joint reaction forces of the glenohumeral joint for a given motion. We used the software OpenSim 3.3 (SimTK, Stanford, CA, USA) and a previously validated shoulder model^7^^,^^23^ to simulate glenohumeral shoulder abduction in the scapular plane between 30° and 80° of glenohumeral abduction. The articulation of the glenohumeral joint was modeled as a ball-and-socket joint as in a relatively constrained version of an anatomical total shoulder replacement. Briefly, the shoulder model consisted of nine muscles spanning the glenohumeral joint with 25 muscle segments. For this study, we investigated 16 muscle segments (deltoid: 6, subscapularis: 3, supraspinatus: 2, infraspinatus: 3, and teres minor: 2). Muscle properties were obtained from a validated model used for similar research questions in which the sum of all deltoid segments compiled to a peak force of 1813 N, whereas rotator cuff muscles achieved an added force of 2079 N (supraspinatus = 364 N, infraspinatus = 945 N, subscapularis = 630 N, and teres minor = 140 N)^33^, ^34^, ^35^ (see Fig. 1). For better comparability with other studies, the maximal generated force of the rotator cuff versus the deltoid had a ratio of approximately 1:1.

In the first step, muscle forces were calculated. To equilibrate the arm weight (3.7 kg) during abduction, a mathematically undetermined interplay of several muscles was needed. The “Static Optimization Tool” was used to calculate the muscle forces at each instant in time, based on performance criteria such as minimizing the sum of squared muscle forces.

In the second step, the joint reaction force was computed. This enabled a relative measurement of stability by using the vector sum muscle forces which were predicted by the model and splitting their components related to the glenoid plane. After obtaining compression (application of pressure toward the glenoid surface) and shear forces (a force applied perpendicular to the glenoid surface), the shoulder instability ratio was calculated by dividing the shear force by the compression force, where a shoulder instability ratio of 0 indicates a fully stable joint. Shoulder joint model coordinate systems were defined as per the recommendations of the International Society of Biomechanics.^41^

We used a shoulder model with a critical shoulder angle (CSA) of 27.2°, representing an average shoulder with concentric osteoarthritis.^3^ Humeral retroversion was 22° and glenoid retroversion 4°, with these variables being unchanged throughout the experiments.

**Figure 2:**
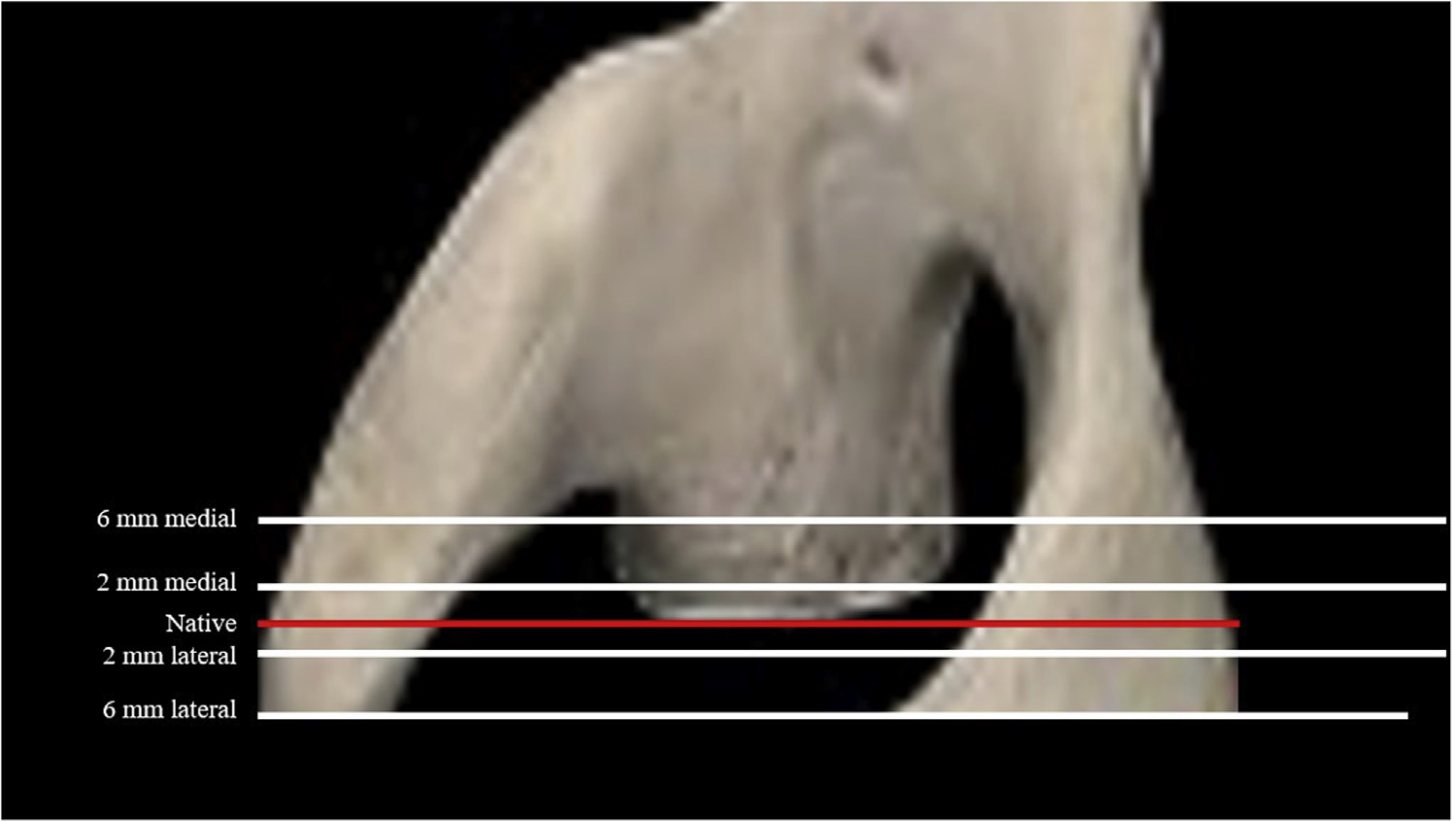
Schematic representation of the displacement of the joint line on the bony model.

The model was then modified to investigate the influence of joint line medialization or lateralization. The center of rotation was moved to five different positions (baseline, 2 mm medialization, 6 mm medialization, 2 mm lateralization, 6 mm lateralization), in line with the Friedman line^16^ for the scapular axis (see Fig. 2).

These changes simulated relevant surgical scenarios. In an unpublished case series of 22 TSA with cemented pegged glenoid components (Zimmer Biomet) in B2 glenoids, we found a medialization of the joint line of about 2 mm. Sabesan et al published, with a 3D computer surgical simulation, that a maximum retroversion of 16° can be corrected on the glenoid side, while maintaining enough glenoid bone stock for glenoid component implantation.^33^ This led to 7 mm of medialization in a standard glenoid (6 mm in the model). Lateralization of the joint line at 6 mm and 2 mm was used to simulate the implantation of a TSA without previous bone loss (thickness of the prosthetic component 6 mm) and those with minimal bone loss, respectively, using a glenoid component of 8 mm thickness. As the center of rotation is moved medially or laterally, the pre-tension of the muscles spanning over the humeral head changes. To compensate for this effect (ie, a longer muscle tendon unit), the algorithm computed more force to contract and bring the muscle tendon unit to its ideal muscle-tendon-length (MTL) force relationship.

Finally, moment arms, muscle forces, joint reaction forces, and MTLs of the deltoid and rotator cuff muscles were analyzed during elevation from 30° to 80° in the scapular plane, for each of the five modified scapular models with altered joint-line position. We reported all data as average values, including each segments of a muscle. Across the motion trial, we measured the outcome ever 2° of glenohumeral abduction.

## Results

### Moment arms of deltoid and rotator cuff muscles

In general, the moment arms of the deltoid muscle segments increased during medialization whereas the moment arms of each rotator cuff muscle decreased. The contrary was observed during lateralization. The effect was most prominent for the anterior deltoid (more than 12% maximal increase or decrease in length) and infraspinatus muscle (more than 3% maximal increase or decrease) (see Table I).

**Table I:** Changes of moment arms.

M DELT	Muscle	2 mm med, %	6 mm med, %	2 mm lat, %	6 mm lat, %
Deltoid	A DELT	4.4	13.1	−4.4	−12.5
	M DELT	−2.9	3.7	−6.4	−8.6
	P DELT	2.4	5.8	−2.6	−7.7
Rotator cuff muscles	ISP	−1.2	−4.0	1.2	3.1
	SSP	−1.2	−4.1	1.1	3.0
	SSC	−0.7	−2.2	0.7	1.7
	TMIN	−0.7	−2.2	0.6	1.7

*A DELT*, anterior deltoid; *M DELT*, middle deltoid; *P DELT*, posterior deltoid; *ISP*, infraspinatus; *SSP*, supraspinatus; SSC, subscapularis; *TMN*, teres minor.

Perceptual increase or decrease in moment arm of shoulder muscles (segments averaged) after incremental medialization or lateralization of glenoid component. Data reported at 60° glenohumeral elevation. Trends are representative for whole curse of glenohumeral elevation from 30° to 80°.

### Muscle forces generated by deltoid and rotator cuff muscles

To position the arm in any degree of elevation a muscle force was generated by the deltoid and rotator cuff muscles.

#### Deltoid muscle

**Figure 3:**
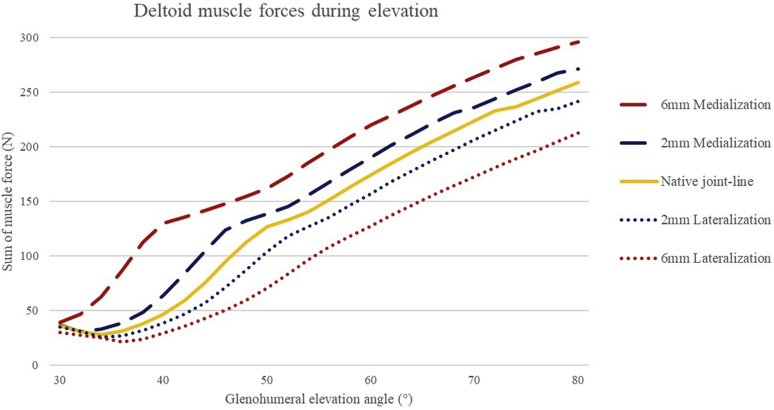
The graphs document that medialization of 6 mm leads to the most important increase (177%) in the deltoid muscle force at around 40° of elevation. These relative changes were decreased if the joint line was lateralized by 6 mm (56%) with the maximal decrease at 50° elevation.

From 30° to 80° of elevation, the deltoid muscle required to generate more force with a medialized joint line to elevate the arm, with a mean difference of 45 N (range, 3-83, SD ± 16) compared with the native joint line. Medialization of 6 mm is associated with a nonlinear increase of deltoid muscle force, especially between 30° and 45° of elevation. A maximal increase in force compared with native joint-line position of 83 N (227%) was found at 40° of elevation. Two mm of medialization leads to a mean increase of deltoid muscle force of 15 N (range 0-30, SD ± 7) compared with the native joint line. In comparison, lateralization of the joint line compared with the native joint line leads to a lower deltoid force required to stabilize the arm between 30° and 80° elevation [2 mm lateralization: −14 N (range 0-26, SD ± 7) and 6 mm lateralization: −38 N (range 3-56, SD ± 17)] (see Fig. 3).

#### Rotator cuff

**Figure 4:**
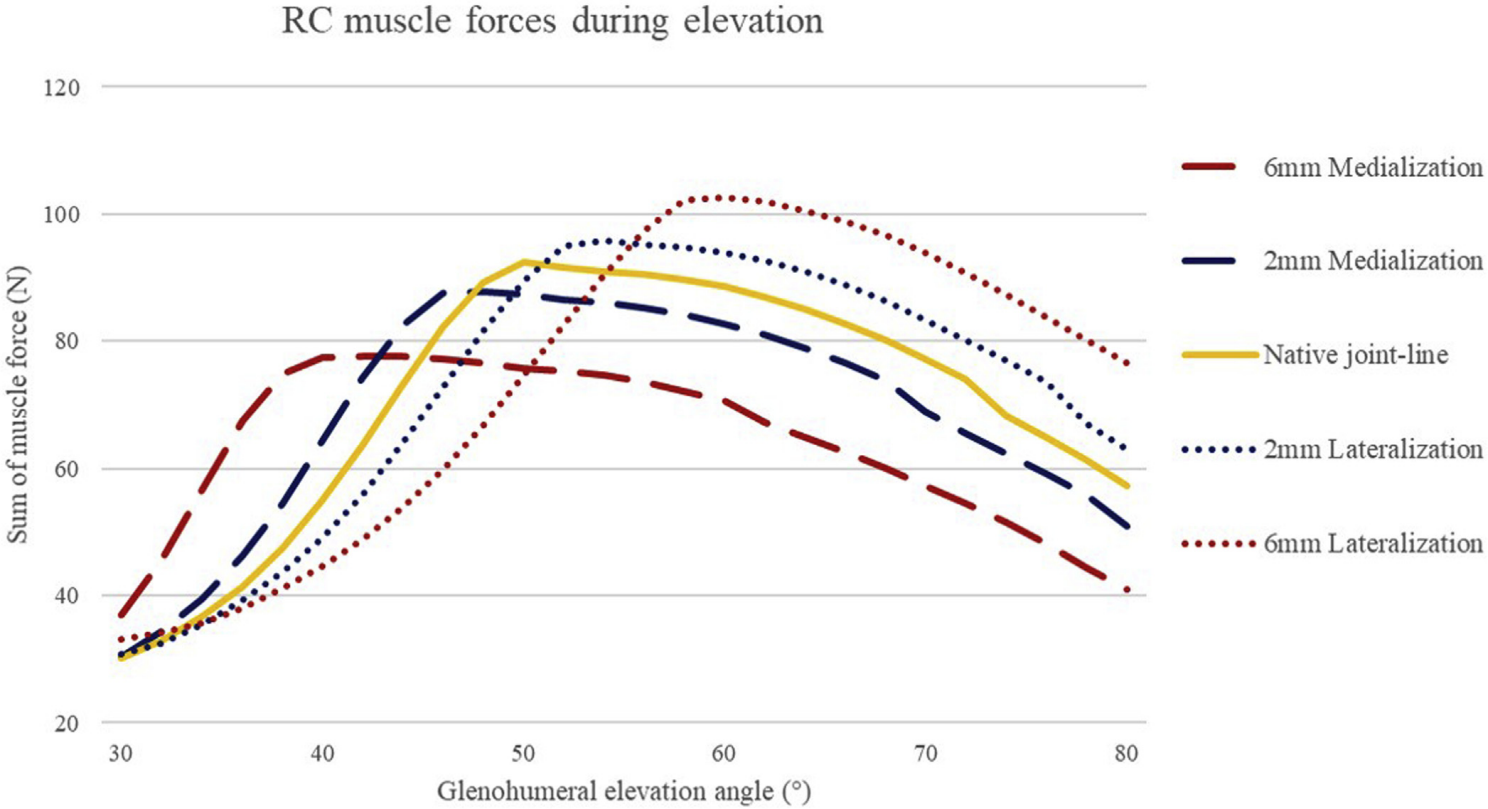
The graphs document that lateralization of 6 mm leads to the most important increase (22%) in the rotator cuff at around 74° of elevation.

With a medialized joint line, rotator cuff muscles force required to stabilize the arm in space are greater with elevation up to 48°, compared with the cuff activity required in the native joint position. However, beyond 48° elevation at the medialized position, the sum of the rotator cuff muscle forces becomes less than that of the native joint. Conversely, lateralization of the joint line leads to higher rotator cuff muscle forces beyond 52° of elevation and to higher absolute values in muscle activity (see Fig. 4).

Over the course of elevation from 30° to 80°, a medial shift of the joint line leads to slightly less total rotator cuff work with a mean difference to the forces generated for the native joint of −7 N for 6 mm (range: −20 to 27, SD ± 16), and mean −2 N (range: −8 to 11, SD ± 6) for 2 mm medialization. On the other hand, lateralization of the joint line required slightly more rotator cuff muscle force compared with the native joint line [2 mm lateralization: mean increase 1 N (range −9 to 9, SD ± 6) and 6 mm lateralization: mean increase 3 N (range −22 to 19, SD ± 14)].

### Joint reaction forces/instability ratio

**Figure 5:**
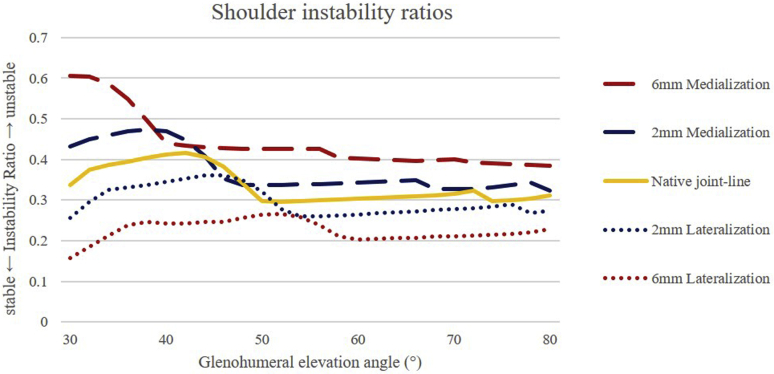
Only with 6 mm medialization of the joint line, an instability ratio >0.6 was reached and therefore required more rotator cuff activity to stabilize the joint.

The instability ratio was found to be higher when the joint line was medialized. A maximum instability ratio of >0.6 was recorded with 6 mm of medialization. An instability ratio of >0.56 has been shown to be the threshold for instability in cadaveric models.^21^^,^^25^ If the instability ratio is greater than 0.56, then greater rotator cuff muscle forces are required to dynamically stabilize the glenohumeral joint, and this was found in our model when the joint line was medialized 6 mm and at a glenohumeral abduction of less than approximately 40 degrees. The instability ratio was less than 0.56 with medialization of 2 mm and for all lateralized joint-line positions, which would indicate no additional rotator cuff force to stabilize the glenohumeral joint (see Fig. 5).

### Muscle-tendon lengths

Deltoid MTLs with lateralization of 2 mm and 6 mm increased an average of 0.7 mm and 4.0 mm, respectively, compared with its original length. While deltoid MTLs decreased by a greater magnitude with joint-line medialization of 2 mm and 6 mm (a decrease of 2.6 mm and 5.8 mm, respectively). For the rotator cuff muscles, a similar effect of muscle tendon shortening during medialization and lengthening was observed. Lateralization of 2 mm and 6 mm resulted in 1.8 and 5.3 mm MTL lengthening on average, respectively. Medialization of 2 mm and 6 mm resulted in MTL shortening of 1.8 mm and 2.9 mm, respectively. The above MTL values were measured at 60° elevation and are representative of data which were measured during whole glenohumeral elevation (not reported).

## Discussion

Our results support the hypothesis that in this 3D computer-based shoulder model, joint line medialization during TSA requires more direct deltoid force to elevate the arm. Our biomechanical model also shows that a medialized joint line is associated with a higher joint instability ratio, as the shear forces generated at the glenohumeral joint were increased. Visually, our computational results can be explained by a more vertical pull of the deltoid, which also transfers less compressive load to the greater tuberosity (Fig. 3). Indirectly, because of the higher instability ratio, more activity of the rotator cuff is needed to compensate and to maintain an instability ratio of 0.55, which was found to be a threshold for joint stability in computer modeling in cadaver models.^21^^,^^38^

Although the additional supraspinatus force to achieve joint stability could not be derived from the present study, an additional supraspinatus force of 51 N was found to stabilize the glenohumeral joint at a glenohumeral abduction angle of 40°. Transferred to our findings, the resulting total supraspinatus force for the 6 mm medialized glenoid will be 100 N (51.4 N additional to stabilize and 48.1 N resulting from the present study) and therefore exceeds the supraspinatus of the native and lateralized joint line. This is in line with previous studies modeling the influence of the CSA on supraspinatus force in the native shoulder.^17^^,^^38^ In these studies, models found that a greater CSA resulted in higher supraspinatus forces, which translates to the present study after TSA is performed, as medialization of the glenoid is in essence, increasing the CSA.

**Figure 6:**
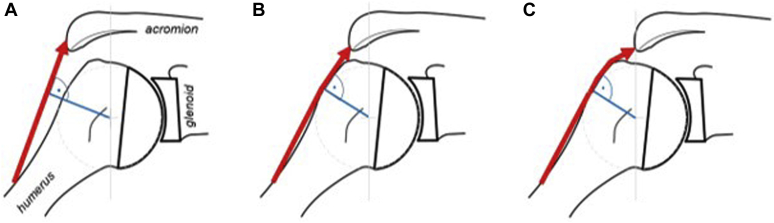
(**A**) medialized, (**B**) native, (**C**) lateralized glenoid. When the joint line is medialized, reduced, or even no wrapping around humerus (tuberculum majus), leading to higher shear forces and no direct compression force on humerus ultimately resulting in higher instability. Shortening of muscles leads to loss of pretension and force. To compensate that effect, more force is necessary to get muscle contraction.

We could show a higher maximal overall activity of the deltoid muscle with medialization of the joint line. These results can be explained by a change in MTLs, which decrease when medializing the joint line and therefore require more force to compensate for this loss of muscle pretension^30^ (see Fig. 6).

Noticeably, the deltoid muscle was more greatly affected by medialization and lateralization than the rotator cuff muscles. This effect can be explained as the moment arm of the rotator cuff muscles is determined by the shape of the humeral head. Contrarily, the deltoid segments are more affected as their moment arms are also dependent on the lateral extension of the acromion (Fig. 6, *A*). This effect only gets limited when the deltoid muscle has contact to the bone where they wrap around the tuberculum majus (eg, in a lateralized condition, Fig. 6, *C*).

Cadaveric studies have shown that implantation of a TSA can lead to lateralization of the glenohumeral joint center by 4.3 ± 3.2 mm^1^; however, this is altered if asymmetrical reaming has to be performed for bone loss or severe retroversion in B2/B3 glenoids.

Regarding the stability of the glenoid component after TSA when correcting glenoid retroversion, biomechanical studies did not detect more implant/bone interface micromotion when correcting retroversion with eccentric reaming (from −15° to 0°) or using an augmented implant at 2000 cycles, but at 10 000 cycles, there was significantly more micromotion in the augmented group.^36^ Furthermore, if retroversion is not corrected, an increase in glenoid component micromotion with retroversion of 10°^13^ and 15°^36^ has been reported. Biomechanical studies comparing reaming and posterior augmenting to correct glenoid retroversion have reported variable results regarding component stress level^22^ or edge displacement.^40^

Minimizing joint lateralization intraoperatively may help to mitigate joint-contact stresses that are believed to contribute to accelerated polyethylene liner wear; however, further research is required to link joint force with component wear rates.^1^

To our knowledge, there is no other study that has biomechanically looked at the effects of medialization and lateralization of the joint line on the loads imposed on the rotator cuff muscles and the deltoid in TSA.

This study has several limitations. First of all, it is a biomechanical investigation with a simulation of muscles and the involved articular surfaces (humerus and glenoid). Capsule and other soft tissues are neglected. Another, for rigid body models common, but nonetheless limiting simplification is the treatment of the glenohumeral joint as an idealized ball-and-socket joint^4^^,^^14^^,^^23^^,^^37^; this disregard translational movement of the glenohumeral joint and is likely to lead to underestimated joint reaction forces. In addition, we used one single model with defined anatomic parameters and therefore the variability in scapula and humeral shape was not considered. The results can therefore only be presented in a descriptive manner without statistical analysis comparing the different models.

## Conclusion

These data support the hypothesis that restoring the premorbid joint line when performing a TSA and avoiding medialization leads to less stresses on the deltoid muscle and reduced force required by the rotator cuff muscles to stabilize the arm in space. In the clinical context, medialization of the joint line by 6 mm from its native position means that the supraspinatus has to apply almost twice as much force and the deltoid muscle nearly 2.7 times more muscle force to stabilize the arm while performing everyday tasks with the arm at 30 to 40 degrees abduction in space.

## Disclaimers:

*Funding:* The authors, their immediate families, and any research foundations with which they are affiliated have not received any financial payments or other benefits from any commercial entity related to the subject of this article.

*Conflicts of interest:* This research received no specific grant from any funding agency in the public, commercial, or not-for-profit sectors.
